# Comparing rapid molecular and culture methods for detecting fungal contamination in healthcare environments

**DOI:** 10.1017/ice.2026.10491

**Published:** 2026-06-18

**Authors:** Bobby G. Warren, Amanda Graves, Aaron Barrett, Guerbine Fils-Aime, Matthew Stiegel, Becky A. Smith, Ilan Schwartz, Deverick J. Anderson

**Affiliations:** 1 Disinfection, Resistance, and Transmission Epidemiology (DiRTE) lab, Durham, NC, USA; 2 Duke Center for Antimicrobial Stewardship and Infection Prevention, Durham, NC, USA; 3 Division of Infectious Diseases, Duke University Medical Center, Durham, NC, USA; 4 Duke Occupational and Environmental Safety Office, Durham, NC, USA

## Abstract

**Background:**

Fungal contamination of healthcare environments is increasingly recognized as a potential contributor to healthcare-associated infections, yet standardized environmental surveillance strategies remain poorly defined. Culture-based methods are widely used but have limitations including low sensitivity and prolonged turnaround time. Molecular approaches such as quantitative PCR (qPCR) may improve detection but have not been extensively evaluated in real-world hospital environments.

**Methods:**

We conducted a longitudinal observational study across three inpatient units at a tertiary academic medical center from September 2023 through June 2024. Environmental samples were collected monthly from patient rooms and shared unit areas, including air, bathroom floors, HVAC components, patient bedrails, and linen storage areas. Samples were analyzed using direct-from-sample 18S qPCR and culture-based detection followed by 18S or ITS sequencing.

**Results:**

Among 742 samples collected, 474 (64%) were positive for fungi by qPCR compared with 213 (29%) by culture (*P* < .01). qPCR showed higher detection rates across most sample types, including air (35% vs 3%), bathroom floors (86% vs 42%), HVAC exhaust vents (72% vs 41%), and patient bedrails (78% vs 10%). Culture methods identified a broader diversity of fungi, including *Talaromyces*, *Candida*, and *Penicillium*, while qPCR detections were dominated by *Malassezia*.

**Conclusions:**

Molecular and culture-based methods provide complementary insights into hospital fungal contamination. qPCR demonstrated greater sensitivity, while culture identified a broader range of viable fungi. Future surveillance strategies may benefit from leveraging qPCR sensitivity using targeted primers for clinically important fungi, reducing the need for broad sequencing and bioinformatic analysis.

## Introduction

Healthcare-associated infections (HAIs) are an ongoing concern; an estimated 700,000 HAIs and 75,000 deaths attributable to HAIs occur annually in the United States.^1,2^ Infection prevention strategies have primarily targeted bacterial pathogens and hand hygiene. However, invasive fungal infections are an increasingly recognized source of morbidity, outbreaks, and excess healthcare costs.^3–5^ Outbreaks of *Candida*, *Aspergillus*, and other molds have highlighted the hospital environment as an important reservoir for transmission, but many aspects of transmission via this reservoir remain unstudied.^6,7^

Despite these growing threats, standardized guidelines or threshold values for environmental fungal surveillance in hospitals do not exist. Conventional culture-based detection methods remain widely used but suffer from limited sensitivity, prolonged turnaround times, and labor-intensive protocols.^8,9^ Newer molecular techniques, such as quantitative PCR (qPCR), have shown promise for rapid, sensitive detection of environmental fungi. However, the performance of qPCR compared to traditional culture methods in real-world hospital settings and its utility for identifying pathogens of greatest clinical importance remain unclear.

Our prior work tested qPCR and various sampling methods in a controlled laboratory setting alongside conventional fungal culture methods.^10^ Based on those findings, we selected sponge sampling as the optimal approach and deployed this method in the real-world hospital environment to more rigorously compare the performance of qPCR to the gold standard of culture-based fungal detection. Thus, the objectives of this study were 1) to establish baseline levels of environmental fungal contamination, 2) to directly compare the performance of qPCR and culture-based methods, and 3) to identify gaps in the detection of clinically relevant fungi for each method.

## Methods

### Study setting and design

We conducted a longitudinal observational study at Duke University Hospital, a 1,062-bed tertiary academic medical center in Durham, North Carolina. The study took place in 3 units in 3 different inpatient bed towers of differing ages: a general medicine unit in bed tower 1 (BT1) opened in 1980; a medical intensive care unit in bed tower 2 (BT2) opened in 2013; and a neurological intensive care unit in bed tower 3 (BT3) opened in 2021. Buildings of differing age were selected to improve generalizability because infrastructure and ventilation characteristics may influence fungal contamination.^11–14^ Throughout the study, patients in all three units were monitored for active invasive fungal infections.

### Sampling protocol

Samples were taken monthly in all three units at two levels: the patient room and the unit. Five patient rooms per unit were prespecified for sampling based on spatial distribution across the unit. Rooms were selected to represent both peripheral and central locations within the unit to capture potential spatial variability in environmental fungal contamination. Patient room-level fomite samples included the bathroom floor, the HVAC intake, and patient bedrails. Additionally, active air samples were taken in each room using an air sampler set to 250 L/min for a total volume of 1,000 L. The sampler was placed centrally within the patient room.

Eight unit-level samples were collected per unit during each sampling event. These included HVAC exhaust vent fomite samples (n = 3), active air samples near HVAC exhaust vents (n = 3), and fomite samples from clean linen storage (n = 1) and soiled linen storage (n = 1).

All fomite samples were collected using Whirl-Pak polyurethane sponges premoistened in neutralizing buffer.^10^ Air samples were collected with the SAS Super 180 air sampler (Bioscience International, Rockville, MD, USA).

### Microbiological methods

**Figure 1.**
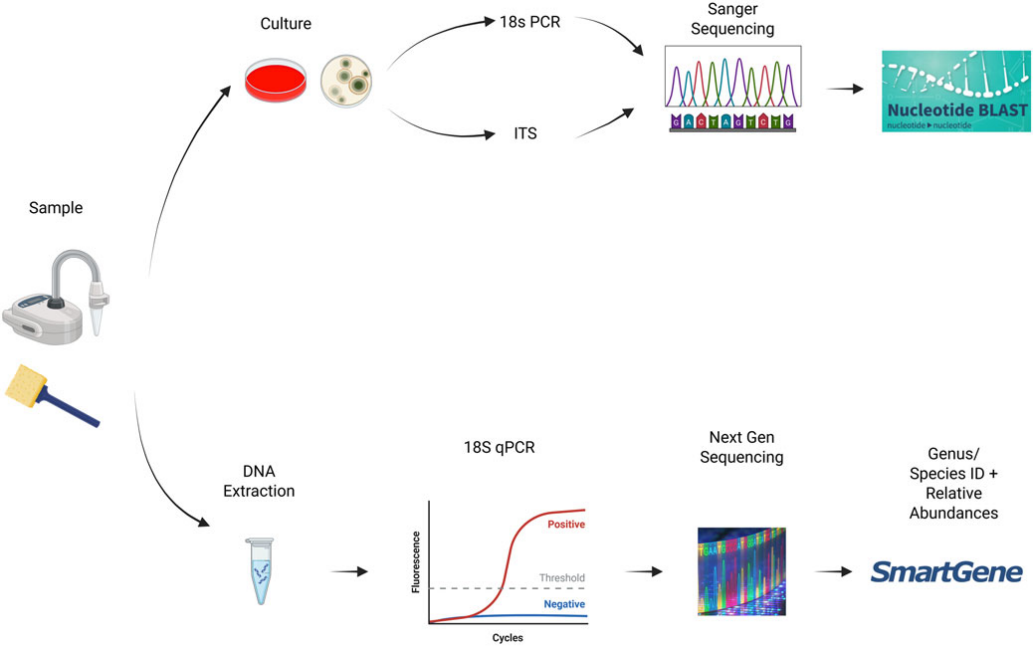
Figure 1 long description. Study workflow for culture-based and direct-from-sample molecular fungal detection.

Environmental samples were evaluated using three fungal detection approaches: direct-from-sample 18S qPCR, culture followed by 18S sequencing (culture–18S), and culture followed by internal transcribed spacer sequencing (culture–ITS) (Figure 1).

For culture-based detection, sponge eluates were plated on both Sabouraud dextrose agar (SDA) plates and HardyCHROM Candida + auris chromogenic media; air samplers were loaded with SDA plates. All study media were incubated at 30°C and 37°C and monitored daily for fungal growth for 7 days. Morphologically distinct colonies underwent 18S and ITS sequencing.

For direct-from-sample 18S qPCR, molecular detection was performed on all samples to assess fungal presence and achieve taxonomic classification via sequencing. DNA was extracted directly from raw environmental sample eluates using the ZymoBIOMICS DNA Miniprep Kit and added to a qPCR master mix containing FungiQuant® primers and FungiQuant® probe, targeting the conserved 18S rRNA gene region. PCR cycling conditions followed published protocols.^9,10,15^

Samples with a qPCR cycle threshold (Ct) value <40 were selected for sequencing. PCR products were purified and prepared for amplicon sequencing using the KAPA HyperPrep kit prior to sequencing on an Illumina NextSeq 1,000 using P1 300 bp paired-end chemistry. Raw reads were analyzed using a microbiome pipeline, allowing for detection of multiple fungal amplicons per sample. Sequencing results were then uploaded and mapped against the SmartGene Integrated Database Network System (IDNS) for 18S to assign phylogenetic identification to the family, genus, or species level.

### Outcomes

The primary outcome was the proportion of environmental samples that tested positive for fungal contamination by each detection method. Secondary outcomes included the level of phylogenetic identification achieved via qPCR, the most common genera and species identified by each detection method, and temporal trends in identified fungal genera over the study period.

### Statistical analysis

All analyses were performed using SAS version 9.4M7. Descriptive statistics were used to summarize detection rates by method, location, and sample type. The χ^2^ test or Fisher’s exact test was used to compare proportions, as appropriate. Trends in fungal genera over time and across units were visualized using heatmaps and bar graphs.

## Results

### Descriptive data

Environmental sampling was conducted monthly from September 2023 through June 2024. Ten monthly sampling events were completed in the three study units. Each scheduled event included 28 samples per unit. Of the resulting 840 potential samples for this study, 742 were collected due to occasional patient refusals. Throughout the study, no patients in study units were identified as having invasive fungal infections.

### Primary outcome

Of the 742 samples obtained, 474 (64%) were positive for targeted fungal pathogens by direct-from-sample 18S qPCR and 213 (29%) by culture-based detection (*P* < .01).

Generally, direct-from-sample 18S qPCR had higher detection rates than culture at the sample location level. Air samples were positive in 72 cases (35%) by qPCR but only 6 (3%) by culture. Similar differences were observed for bathroom floors (113[86%] vs 56[42%]), HVAC exhaust vents (160[72%] vs 90[41%]), and patient bedrails (93[78%] vs 12[10%]) (all *P* < .01). In contrast, results were similar in clean and dirty linen storage areas: 13(43%) vs 9(30%) for clean linen (*P* = .13), and 23(77%) vs 27(90%) for dirty linen (*P* = .05). Despite overall differences in sensitivity, both detection methods consistently identified bathroom floors, dirty linen storage, and HVAC exhaust vents as the most frequently contaminated sites (Table 1).

**Table 1.** Hospital environmental samples positive for fungi via direct-from-sample 18S qPCR and culture detection methods overall and by sample location and study unit Table 1 long description.

	qPCR n (%)	Culture n (%)	*P*-value
Overall (N = 742)	474 (64)	213 (29)	<.01
Air (N = 208)	72 (35)	6 (3)	<.01
Bathroom floor (N = 132)	113 (86)	56 (42)	<.01
Clean linen storage (N = 30)	13 (43)	9 (30)	.13
Dirty linen storage (N = 30)	23 (77)	27 (90)	.05
HVAC exhaust vent (N = 222)	160 (72)	90 (41)	<.01
Patient bedrails (N = 120)	93 (78)	12 (10)	<.01
Bed tower 1 (N = 253)	175 (69)	96 (38)	<.01
Air (N = 68)	26 (38)	1 (1)	<.01
Bathroom floor (N = 47)	38 (81)	22 (47)	<.01
Clean linen storage (N = 10)	4 (40)	5 (50)	<.01
Dirty linen storage (N = 10)	10 (100)	10 (100)	1
HVAC exhaust vent (N = 77)	66 (86)	46 (60)	<.01
Patient bedrails (N = 41)	31 (76)	12 (29)	<.01
Bed tower 2 (N = 239)	145 (61)	61 (26)	<.01
Air (N = 69)	18 (26)	1 (1)	<.01
Bathroom floor (N = 40)	39 (98)	17 (43)	<.01
Clean linen storage (N = 10)	5 (50)	1 (10)	<.01
Dirty linen storage (N = 10)	6 (60)	10 (100)	.03
HVAC exhaust vent (N = 71)	46 (65)	28 (39)	<.01
Patient bedrails (N = 39)	31 (79)	4 (10)	<.01
Bed tower 3 (N = 250)	154 (62)	56 (22)	<.01
Air (N = 71)	28 (39)	4 (6)	<.01
Bathroom floor (N = 45)	36 (80)	17 (38)	<.01
Clean linen storage (N = 10)	4 (40)	3 (30)	.51
Dirty linen storage (N = 10)	7 (70)	7 (70)	1
HVAC exhaust vent (N = 74)	48 (65)	16 (22)	<.01
Patient bedrails (N = 40)	31 (78)	9 (23)	<.01

At the study unit and bed tower level, direct-from-sample 18S qPCR’s higher detection rate persisted. In BT1, BT2, and BT3, direct-from-sample 18S qPCR identified fungal contamination in 175(69%), 145(61%), and 154(62%) of samples, respectively. In contrast, culture-based detection yielded 96 (38%), 61 (26%), and 56 (22%) positive samples (all *P* < .01). Contamination rates correlated with building age for both detection methods, with the highest levels observed in BT1 (the oldest), followed by BT2, then BT3 (the newest) (qPCR *P* = .05; culture *P* < .01). Notably, BT1 also showed the smallest difference in detection rates between methods.

### Level of phylogenetic identification via direct-from-sample 18S qPCR

Among the 474 direct-from-sample 18S qPCR-positive samples, a total of 787 fungal detections were resolved to at least the family, genus, or species level. Of these, 106 (13%) were identified to the family level, 145 (18%) to the genus level, and 536 (68%) to the species level (Table 2).

**Table 2.** Level of phylogenetic identification via direct-from-sample 18S qPCR of hospital environmental samples positive for fungal presence Table 2 long description.

	Family n (%)	Genus n (%)	Species n (%)
Overall (N = 787)	106 (13)	145 (18)	536 (68)
Air (N = 118)	18 (15)	23 (19)	77 (65)
Bathroom floor (N = 177)	26 (15)	28 (16)	123 (69)
Clean linen storage (N = 25)	3 (12)	7 (28)	15 (60)
Dirty linen storage (N = 25)	3 (12)	13 (52)	9 (36)
HVAC exhaust vent (N = 242)	46 (19)	44 (18)	152 (63)
Patient bedrails (N = 200)	10 (5)	30 (15)	160 (80)
Bed tower 1 (N = 303)	52 (17)	60 (20)	191 (63)
Air (N = 42)	6 (14)	9 (21)	27 (64)
Bathroom floor (N = 76)	12 (16)	9 (12)	55 (72)
Clean linen storage (N = 8)	1 (12)	4 (50)	3 (38)
Dirty linen storage (N = 21)	3 (14)	12 (57)	6 (29)
HVAC exhaust vent (N = 89)	23 (26)	15 (17)	51 (57)
Patient bedrails (N = 67)	7 (10)	11 (16)	49 (73)
Bed tower 2 (N = 229)	34 (15)	38 (17)	157 (69)
Air (N = 31)	6 (19)	3 (10)	22 (71)
Bathroom floor (N = 46)	8 (17)	10 (22)	28 (61)
Clean linen storage (N = 13)	2 (15)	3 (23)	8 (62)
Dirty linen storage (N = 1)	0 (0)	0 (0)	1 (100)
HVAC exhaust vent (N = 77)	17 (22)	11 (14)	49 (64)
Patient bedrails (N = 61)	1 (2)	11 (18)	49 (80)
Bed tower 3 (N = 255)	20 (8)	47 (18)	188 (74)
Air (N = 45)	6 (13)	11 (24)	28 (62)
Bathroom floor (N = 55)	6 (11)	9 (16)	40 (73)
Clean linen storage (N = 4)	0 (0)	0 (0)	4 (100)
Dirty linen storage (N = 3)	0 (0)	1 (33)	2 (67)
HVAC exhaust vent (N = 76)	6 (8)	18 (24)	52 (68)
Patient bedrails (N = 72)	2 (3)	8 (11)	62 (86)

At the sample location level, this pattern remained generally consistent. Patient bedrails showed the highest proportion of species-level identifications at 160 (80%), followed by bathroom floors with 123 (69%) and air samples with 77 (65%). Genus-level identifications ranged from 15% (bedrails) to 52% (dirty linen storage), with dirty linen storage also having the lowest species-level resolution at only 9 (36%).

By study unit, 191 of 303 fungi (63%) from BT1, 157 of 229 (69%) from BT2, and 188 of 255 (74%) from BT3 were identified at the species level. The overall comparison across all three units was not statistically significant (*P* = .13). Identification patterns by sample location within each unit mirrored the overall trends, with patient bedrails and HVAC exhaust vents consistently yielding high species-level identification, while dirty linen storage lagged across all units.

### Most common genera identified by detection methods

**Figure 2.**
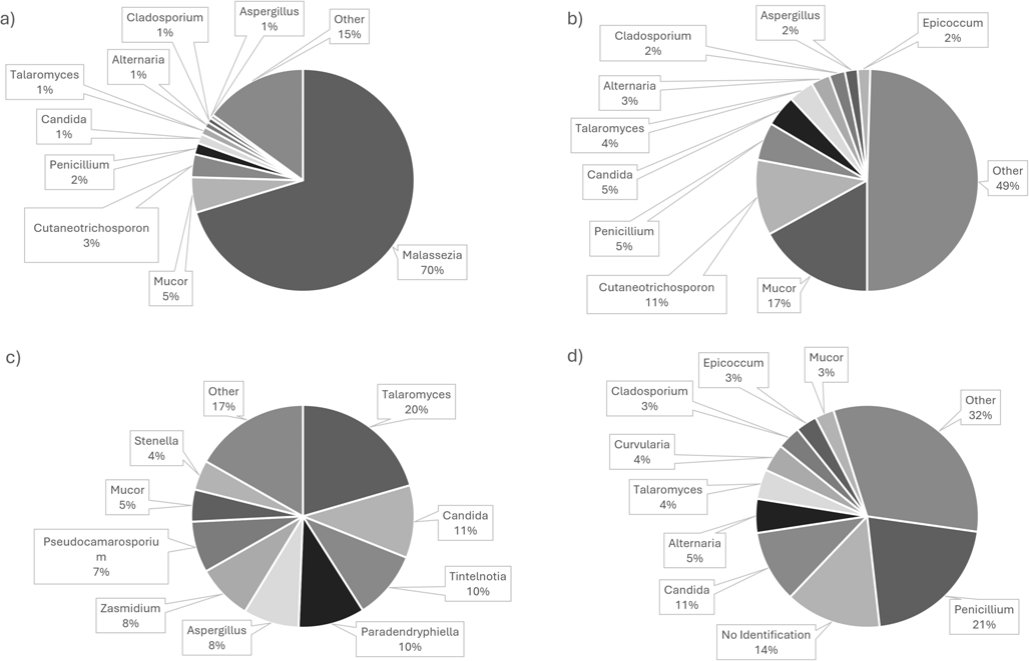
Figure 2 long description. Top 10 genera identified in fungal-positive hospital environmental samples by detection method. (a) Direct-from-sample 18S qPCR, (b) direct-from-sample 18S qPCR excluding Malassezia spp., (c) culture followed by 18S, (d) culture followed by ITS.

Using direct-from-sample 18S qPCR, the most frequently detected genus was *Malassezia*, accounting for 507 (68%) of all identified genera. Although *Malassezia* is a common environmental fungus, it is typically of less clinical concern. When excluded from analysis, *Mucor* emerged as the most prevalent clinically relevant genus (37 detections, 5%), followed by *Cutaneotrichosporon* (24, 3%) and *Penicillium* (12, 2%) (Table 3). The overall distribution of the ten most frequently detected genera across detection methods is shown in Figure 2.

**Table 3.** Most common genera identified in hospital environmental samples positive for fungi via direct-from-sample 18S qPCR and culture detection Table 3 long description.

	18s qPCR genus n (%)			18s qPCR sans *Malassezia spp.* genus n (%)			Culture via 18s genus n (%)			Culture via ITS genus n (%)		
	1st Most common	2nd Most common	3rd Most common	1st Most common	2nd Most common	3rd Most common	1st Most common	2nd Most common	3rd Most common	1st Most common	2nd Most common	3rd Most common
Overall	Malassezia 507 (68)	Mucor 37 (5)	Cutaneotrichosporon 24 (3)	Mucor 37 (5)	Cutaneotrichosporon 24 (3)	Penicillium 12 (2)	Talaromyces 66 (9)	Candida 34 (5)	Tintelnotia 32 (4)	Penicillium 68 (9)	No Identification 45 (6)	Candida 34 (5)
Air	Malassezia 71 (34)	Mucor 5 (2)	Penicillium 5 (2)	Mucor 5 (2)	Penicillium 5 (2)	Cutaneotrichosporon 3 (1)	Talaromyces 4 (2)	Alternaria 1 (1)	Aspergillus 1 (1)	Penicillium 5 (2)	Alternaria 1 (1)	–
Bathroom floor	Malassezia 124 (94)	Cutaneotrichosporon 5 (4)	Symmetrospora 4 (3)	Cutaneotrichosporon 5 (4)	Symmetrospora 4 (3)	Naganishia 3 (2)	Candida 13 (10)	Talaromyces 9 (7)	Tintelnotia 9 (7)	Candida 13 (10)	Penicillium 10 (8)	No Identification 10 (8)
Clean linen storage	Malassezia 13 (43)	Mucor 3 (10)	Baeospora 1 (3)	Mucor 3 (10)	Baeospora 1 (3)	Botryosphaeria 1 (3)	Talaromyces 4 (13)	Mucor 3 (10)	Zasmidium 2 (7)	Penicillium 4 (13)	No Identification 4 (13)	Trichoderma 2 (7)
Dirty linen storage	Mucor 7 (23)	Torulaspora 7 (23)	Candida 2 (7)	Mucor 7 (23)	Torulaspora 7 (23)	Candida 2 (7)	Talaromyces 13 (43)	Candida 8 (27)	Mucor 8 (27)	Penicillium 12 (40)	Candida 8 (27)	Mucor 8 (27)
HVAC exhaust vent	Malassezia 132 (59)	Mucor 13 (6)	Penicillium 7 (3)	Mucor 13 (6)	Penicillium 7 (3)	Cutaneotrichosporon 6 (3)	Talaromyces 30 (14)	Paradendryphiella 21 (9)	Tintelnotia 20 (9)	Penicillium 31 (14)	No Identification 23 (10)	Curvularia 19 (9)
Patient bedrails	Malassezia 167 (100)	Mucor 6 (5)	Cutaneotrichosporon 4 (3)	Mucor 6 (5)	Cutaneotrichosporon 4 (3)	Anguillospora 2 (2)	Candida 7 (6)	Talaromyces 6 (3)	Zasmidium 3 (3)	Candida 7 (6)	Penicillium 6 (5)	No Identification 4 (3)
Bed tower 1	Malassezia 204 (81)	Mucor 13 (5)	Aspergillus 6 (2)	Mucor 13 (5)	Aspergillus 6 (2)	Cutaneotrichosporon 6 (2)	Talaromyces 27 (11)	Tintelnotia 21 (8)	Candida 15 (6)	Penicillium 27 (11)	No Identification 22 (9)	Epicoccum 17 (7)
Air	Malassezia 28 (41)	Aspergillus 2 (3)	Penicillium 2 (3)	Aspergillus 2 (3)	Penicillium 2 (3)	Hydnoporia 1 (1)	Aspergillus 1 (1)	–	–	Penicillium 1 (1)	–	–
Bathroom floor	Malassezia 56 (100)	Cutaneotrichosporon 3 (6)	Symmetrospora 3 (6)	Cutaneotrichosporon 3 (6)	Symmetrospora 3 (6)	Chaetosphaeria 2 (4)	Tintelnotia 6 (13)	Zasmidium 5 (11)	Candida 4 (9)	Epicoccum 5 (11)	No Identification 5 (11)	Candida 4 (9)
Clean linen storage	Malassezia 3 (30)	Mucor 2 (20)	Trichoderma 1 (10)	Mucor 2 (20)	Trichoderma 1 (10)	Tyromyces 1 (10)	Mucor 2 (20)	Talaromyces 2 (20)	Aspergillus 1 (10)	Penicillium 2 (20)	Aspergillus 1 (10)	Mucor 1 (10)
Dirty linen storage	Mucor 7 (70)	Torulaspora 7 (70)	Candida 2 (20)	Mucor 7 (70)	Torulaspora 7 (70)	Candida 2 (20)	Mucor 8 (80)	Candida 5 (50)	Talaromyces 5 (50)	Mucor 8 (80)	Candida 5 (50)	Penicillium 5 (50)
HVAC exhaust vent	Malassezia 51 (66)	Mucor 4 (5)	Bradymyces 2 (3)	Mucor 4 (5)	Bradymyces 2 (3)	Clathrospora 1 (1)	Talaromyces 17 (22)	Tintelnotia 15 (19)	Aspergillus 10 (13)	Penicillium 15 (19)	No Identification 14 (18)	Epicoccum 12 (16)
Patient bedrails	Malassezia 49 (100)	Cutaneotrichosporon 3 (7)	Mucor 3 (7)	Cutaneotrichosporon 3 (7)	Mucor 3 (7)	Taphrina 2 (5)	Zasmidium 3 (7)	Candida 2 (5)	Talaromyces 2 (5)	Cladosporium 3 (7)	Candida 2 (5)	Penicillium 2 (5)
Bed tower 2	Malassezia 142 (59)	Mucor 9 (4)	Parasarocladium 5 (2)	Mucor 9 (4)	Parasarocladium 5 (2)	Poitrasia 4 (2)	Talaromyces 18 (8)	Paradendryphiella 12 (5)	Candida 11 (5)	Penicillium 19 (8)	Curvularia 13 (5)	Candida 11 (5)
Air	Malassezia 15 (22)	Mucor 2 (3)	Bradymyces 1 (1)	Mucor 2 (3)	Bradymyces 1 (1)	Clathrospora 1 (1)	Alternaria 1 (1)	–	–	Alternaria 1 (1)	–	–
Bathroom floor	Malassezia 32 (80)	Parasarocladium 3 (8)	Poitrasia 2 (5)	Parasarocladium 3 (8)	Poitrasia 2 (5)	Pseudocercospora 2 (5)	Candida 7 (18)	Paradendryphiella 3 (8)	Talaromyces 3 (8)	Candida 7 (18)	No Identification 4 (10)	Curvularia 3 (8)
Clean linen storage	Malassezia 7 (10)	Baeospora 1 (10)	Cutaneotrichosporon 1 (10)	Baeospora 1 (10)	Cutaneotrichosporon 1 (10)	Mucor 1 (10)	Talaromyces 1 (10)	–	–	Penicillium 1 (10)	–	–
Dirty linen storage	Filobasidium 1 (10)	–	–	Filobasidium 1 (10)	–	–	Talaromyces 6 (60)	Stenella 2 (20)	Alternaria 1 (10)	Penicillium 5 (50)	Cladosporium 2 (20)	Alternaria 1 (10)
HVAC exhaust vent	Malassezia 32 (45)	Cutaneotrichosporon 4 (6)	Penicillium 3 (4)	Cutaneotrichosporon 4 (6)	Penicillium 3 (4)	Poitrasia 3 (4)	Paradendryphiella 8 (11)	Pseudocamarosporium 7 (10)	Talaromyces 7 (10)	Curvularia 9 (13)	Penicillium 9 (13)	Pseudopithomyces 7 (10)
Patient bedrails	Malassezia 56 (100)	Baeospora 1 (3)	Clavispora 1 (3)	Baeospora 1 (3)	Clavispora 1 (3)	Penicillium 1 (3)	Candida 2 (5)	Botryotrichum 1 (3)	Talaromyces 1 (3)	Candida 2 (5)	Dinemasporium 1 (3)	Penicillium 1 (3)
Bed tower 3	Malassezia 178 (71)	Mucor 15 (6)	Penicillium 3 (1)	Mucor 15 (6)	Penicillium 3 (1)	Cutaneotrichosporon 2 (1)	Cutaneotrichosporon 3 (1)	Candida 8 (3)	Paradendryphiella 7 (3)	Penicillium 22 (9)	No Identification 12 (5)	Candida 8 (3)
Air	Malassezia 28 (39)	Mucor 3 (4)	Cutaneotrichosporon 2 (3)	Mucor 3 (4)	Cutaneotrichosporon 2 (3)	Penicillium 2 (3)	Talaromyces 4 (6)	–	–	Penicillium 4 (6)	–	–
Bathroom floor	Malassezia 36 (80)	Mucor 2 (4)	Neophaeococcomyces 2 (4)	Mucor 2 (4)	Neophaeococcomyces 2 (4)	Aspergillus 1 (2)	Talaromyces 5 (11)	Candida 2 (4)	Paradendryphiella 2 (4)	Penicillium 5 (11)	Aspergillus 2 (4)	Candida 2 (4)
Clean linen storage	Malassezia 3 (30)	Botryosphaeria 1 (10)	–	Botryosphaeria 1 (10)	–	–	Zasmidium 2 (20)	Mucor 1 (10)	Pseudocamarosporium 1 (10)	No Identification 3 (30)	Cladosporium 1 (10)	Penicillium 1 (10)
Dirty linen storage	Clavispora 1 (10)	Kurtzmaniella 1 (10)	Rhizopus 1 (10)	Clavispora 1 (10)	Kurtzmaniella 1 (10)	Rhizopus 1 (10)	Aspergillus 2 (20)	Candida 2 (20)	Talaromyces 2 (20)	No Identification 3 (30)	Aspergillus 2 (20)	Candida 2 (20)
HVAC exhaust vent	Malassezia 49 (66)	Mucor 7 (9)	Penicillium 3 (4)	Mucor 7 (9)	Penicillium 3 (4)	Ophiostoma 2 (3)	Talaromyces 6 (8)	Paradendryphiella 5 (7)	Aspergillus 2 (3)	Penicillium 7 (9)	Curvularia 4 (5)	No Identification 3 (4)
Patient bedrails	Malassezia 62 (100)	Mucor 3 (8)	Poitrasia 2 (5)	Mucor 3 (8)	Poitrasia 2 (5)	Anguillospora 1 (3)	Candida 3 (8)	Talaromyces 3 (8)	Ambrosiella 1 (3)	Penicillium 3 (8)	No Identification 2 (5)	–

Culture-18s identified *Talaromyces* most frequently, with 66 detections (9%), followed by *Candida* (34, 5%) and *Tintelnotia* (32, 4%). Culture-ITS revealed *Penicillium* as the most common genus at 68 detections (9%), with a considerable number of samples (45, 6%) resulting in “No Identification.” Thereafter, *Candida* was again prominent (34, 5%), along with *Talaromyces* and *Curvularia*.

The distribution of genera across sample locations generally followed the same trends seen across detection methods. *Mucor* was the most frequently identified clinically relevant genus via direct-from-sample 18S qPCR (excluding *Malassezia*), *Talaromyces* dominated culture-18S results, and *Penicillium* was the most common genus in culture-ITS results. Notable exceptions included *Cutaneotrichosporon*, which was the most frequent genus by direct-from-sample 18S qPCR on bathroom floors, and *Candida*, which was the most frequent on bathroom floors and patient bedrails via both culture methods.

By study unit, the same general detection patterns were observed. *Mucor* was the most common genus via direct-from-sample 18S qPCR across all three units. *Talaromyces* was predominant in culture-18S, while *Penicillium* dominated culture-ITS identifications. Notably, BT1 had the highest detection of clinically relevant fungi such as *Aspergillus*, *Penicillium*, and *Mucor*.

### Most common species identified by detection methods

Species identification by direct-from-sample 18S qPCR was dominated by Malassezia species, particularly *M. restricta* (232, 31%) and *M. globosa* (141, 19%). Excluding Malassezia, qPCR most commonly detected *Penicillium paradoxum* (12, 2%), followed by *Cutaneotrichosporon haglerorum* (8, 1%) and *Clathrospora diplospora* (4, 1%), reflecting a narrower range of detections beyond commensal yeasts.

Culture-18S yielded a broader range of clinically significant fungi. *Talaromyces wortmannii* was the most frequently detected species (63, 8%), followed by *Tintelnotia destructans* (32, 4%) and *Paradendryphiella arenariae* (30, 4%). Other relevant pathogens included *Candida glabrata*, *Mucor janssenii*, and *Aspergillus niger*.

The most common species for culture-ITS was *Penicillium fuscoglaucum* (40, 5%), with *Epicoccum phragmospora* (21, 3%) and unidentified fungi (45, 6%) also appearing frequently.

At the sample location level, species-level detection patterns varied considerably across both locations and detection methods. Air samples showed differing profiles depending on the method used, while bathroom floors and patient bedrails also demonstrated inconsistent species detection between qPCR and culture approaches. HVAC exhaust vents showed somewhat greater agreement across methods, with several taxa detected by both molecular and culture techniques. Overall, species-level detections were heterogeneous across sample types without a consistent dominant species across all environments.

Across study units, direct-from-sample 18S qPCR identified a mix of Malassezia and other environmental fungi without clear dominance by any non-commensal taxa. Culture results varied by unit and can be seen in Table 4.

**Table 4.** Most common species identified in hospital environmental samples positive for fungi via direct-from-sample 18S qPCR and culture detection methods Table 4 long description.

	18s qPCR species n (%)			18s qPCR sans *Malassezia spp.* species n (%)			Culture via 18s species n (%)			Culture via ITS species n (%)		
	1st Most common	2nd Most common	3rd Most common	1st Most common	2nd Most common	3rd Most common	1st Most common	2nd Most common	3rd Most common	1st Most common	2nd Most common	3rd Most common
Overall	Malassezia restricta 232 (31)	Malassezia globosa 141 (19)	Penicillium paradoxum 12 (2)	Penicillium paradoxum 12 (2)	Cutaneotrichosporon haglerorum 8 (1)	Clathrospora diplospora 4 (1)	Talaromyces wortmannii 63 (8)	Tintelnotia destructans 32 (4)	Paradendryphiella arenariae 30 (4)	No Identification 45 (6)	Penicillium fuscoglaucum 40 (5)	Epicoccum phragmospora 21 (3)
Air	Malassezia restricta 36 (17)	Malassezia globosa 22 (11)	Penicillium paradoxum 5 (2)	Penicillium paradoxum 5 (2)	Cutaneotrichosporon haglerorum 3 (1)	Botryobasidium botryosum 1 (0)	Talaromyces wortmannii 4 (2)	Alternaria atra 1 (0)	Aspergillus niger 1 (0)	Penicillium fimorum 2 (1)	Penicillium fuscoglaucum 2 (1)	Alternaria alstroemeriae 1 (0)
Bathroom floor	Malassezia restricta 65 (49)	Malassezia globosa 39 (30)	Cutaneotrichosporon cutaneum 3 (2)	Cutaneotrichosporon cutaneum 3 (2)	Poitrasia circinans 3 (2)	Chaetosphaeria ciliata 2 (2)	Talaromyces wortmannii 9 (7)	Tintelnotia destructans 9 (7)	Zasmidium cellare 8 (6)	No Identification 10 (8)	Epicoccum phragmospora 8 (6)	Candida glabrata 6 (5)
Clean linen storage	Malassezia restricta 7 (23)	Malassezia globosa 3 (10)	Baeospora myosura 1 (3)	Baeospora myosura 1 (3)	Botryosphaeria agaves 1 (3)	Cutaneotrichosporon haglerorum 1 (3)	Talaromyces wortmannii 4 (13)	Mucor janssenii 2 (7)	Zasmidium cellare 2 (7)	Penicillium fuscoglaucum 4 (13)	No Identification 4 (13)	Aspergillus awamori 1 (3)
Dirty linen storage	Candida bohioensis 2 (7)	Clavispora lusitaniae 2 (7)	Filobasidium uniguttulatum 2 (7)	Candida bohioensis 2 (7)	Clavispora lusitaniae 2 (7)	Filobasidium uniguttulatum 2 (7)	Talaromyces wortmannii 12 (40)	Mucor janssenii 8 (27)	Candida glabrata 5 (17)	Penicillium fuscoglaucum 9 (30)	Mucor circinelloides 7 (23)	Candida glabrata 5 (17)
HVAC exhaust vent	Malassezia restricta 74 (33)	Malassezia globosa 40 (18)	Penicillium paradoxum 7 (3)	Penicillium paradoxum 7 (3)	Cutaneotrichosporon haglerorum 5 (2)	Clathrospora diplospora 4 (2)	Talaromyces wortmannii 28 (13)	Paradendryphiella arenariae 21 (9)	Tintelnotia destructans 20 (9)	No Identification 23 (10)	Penicillium fuscoglaucum 17 (8)	Epicoccum phragmospora 13 (6)
Patient bedrails	Malassezia restricta 86 (72)	Malassezia globosa 59 (49)	Cutaneotrichosporon haglerorum 4 (3)	Cutaneotrichosporon haglerorum 4 (3)	Anguillospora rubescens 2 (2)	Clavispora lusitaniae 2 (2)	Talaromyces wortmannii 6 (5)	Candida glabrata 3 (3)	Zasmidium cellare 3 (3)	Penicillium fuscoglaucum 5 (4)	No Identification 4 (3)	Candida glabrata 3 (3)
Bed tower 1	Malassezia restricta 87 (34)	Malassezia globosa 50 (20)	Cutaneotrichosporon haglerorum 3 (1)	Cutaneotrichosporon haglerorum 3 (1)	Cutaneotrichosporon cutaneum 2 (1)	Graphiola phoenicis 2 (1)	Talaromyces wortmannii 25 (10)	Tintelnotia destructans 21 (8)	Aspergillus niger 14 (6)	No Identification 22 (9)	Penicillium fuscoglaucum 20 (8)	Epicoccum phragmospora 17 (7)
Air	Malassezia globosa 14 (21)	Malassezia restricta 9 (13)	Penicillium paradoxum 2 (3)	Penicillium paradoxum 2 (3)	Hydnoporia olivacea 1 (1)	Neophaeococcomyces oklahomaensis 1 (1)	Aspergillus niger 1 (1)	–	–	Penicillium sumatraense 1 (1)	–	–
Bathroom floor	Malassezia restricta 29 (62)	Malassezia globosa 20 (43)	Chaetosphaeria ciliata 2 (4)	Chaetosphaeria ciliata 2 (4)	Cutaneotrichosporon cutaneum 2 (4)	Graphiola phoenicis 2 (4)	Tintelnotia destructans 6 (13)	Zasmidium cellare 5 (11)	Candida glabrata 3 (6)	Epicoccum phragmospora 5 (11)	No speciation 5 (11)	Candida glabrata 3 (6)
Clean linen storage	Malassezia globosa 1 (10)	Malassezia restricta 1 (10)	Tyromyces chioneus 1 (10)	Tyromyces chioneus 1 (10)	–	–	Mucor janssenii 2 (20)	Talaromyces wortmannii 2 (20)	Aspergillus niger 1 (10)	Penicillium fuscoglaucum 2 (20)	Aspergillus awamori 1 (10)	Mucor circinelloides 1 (10)
Dirty linen storage	Candida bohioensis 2 (20)	Clavispora lusitaniae 1 (10)	Filobasidium uniguttulatum 1 (10)	Candida bohioensis 2 (20)	Clavispora lusitaniae 1 (10)	Filobasidium uniguttulatum 1 (10)	Mucor janssenii 8 (80)	Talaromyces wortmannii 5 (50)	Candida glabrata 4 (40)	Mucor circinelloides 7 (70)	Candida glabrata 4 (40)	Penicillium fuscoglaucum 4 (40)
HVAC exhaust vent	Malassezia restricta 31 (40)	Malassezia globosa 14 (18)	Bradymyces oncorhynchi 2 (3)	Bradymyces oncorhynchi 2 (3)	Clathrospora diplospora 1 (1)	Cutaneotrichosporon haglerorum 1 (1)	Talaromyces wortmannii 15 (19)	Tintelnotia destructans 15 (19)	Aspergillus niger 10 (13)	No Identification 14 (18)	Epicoccum phragmospora 12 (16)	Penicillium fuscoglaucum 12 (16)
Patient bedrails	Malassezia restricta 27 (66)	Malassezia globosa 16 (39)	Cutaneotrichosporon haglerorum 3 (7)	Cutaneotrichosporon haglerorum 3 (7)	Acremonium minutisporum 1 (2)	Anguillospora rubescens 1 (2)	Zasmidium cellare 3 (7)	Talaromyces wortmannii 2 (5)	Aspergillus niger 1 (2)	Cladosporium montecillanum 2 (5)	Penicillium fuscoglaucum 2 (5)	No Identification 2 (5)
Bed tower 2	Malassezia restricta 74 (31)	Malassezia globosa 41 (17)	Cutaneotrichosporon haglerorum 3 (1)	Cutaneotrichosporon haglerorum 3 (1)	Penicillium paradoxum 3 (1)	Poitrasia circinans 3 (1)	Talaromyces wortmannii 17 (7)	Paradendryphiella arenariae 12 (5)	Pseudocamarosporium piceae 9 (4)	No Identification 11 (5)	Penicillium fuscoglaucum 8 (3)	Candida glabrata 6 (3)
Air	Malassezia restricta 12 (17)	Malassezia globosa 3 (4)	Bradymyces oncorhynchi 1 (1)	Bradymyces oncorhynchi 1 (1)	Clathrospora diplospora 1 (1)	Cutaneotrichosporon haglerorum 1 (1)	Alternaria atra 1 (1)	–	–	Alternaria alstroemeriae 1 (1)	–	–
Bathroom floor	Malassezia restricta 16 (40)	Malassezia globosa 9 (23)	Poitrasia circinans 2 (5)	Poitrasia circinans 2 (5)	Pseudocercospora fijiensis 2 (5)	Cutaneotrichosporon cutaneum 1 (3)	Candida glabrata 3 (8)	Paradendryphiella arenariae 3 (8)	Talaromyces wortmannii 3 (8)	No Identification 4 (10)	Candida glabrata 3 (8)	Candida krusei 2 (5)
Clean linen storage	Malassezia restricta 3 (30)	Malassezia globosa 2 (20)	Baeospora myosura 1 (10)	Baeospora myosura 1 (10)	Cutaneotrichosporon haglerorum 1 (10)	Plagiostoma euphorbiae 1 (10)	Talaromyces wortmannii 1 (10)	–	–	Penicillium fuscoglaucum 1 (10)	–	–
Dirty linen storage	Filobasidium uniguttulatum 1 (10)	–	–	Filobasidium uniguttulatum 1 (10)	–	–	Talaromyces wortmannii 5 (50)	Stenella araguata 2 (20)	Alternaria atra 1 (10)	Penicillium fuscoglaucum 4 (40)	Cladosporium endophyticum 2 (20)	Alternaria alstroemeriae 1 (10)
HVAC exhaust vent	Malassezia restricta 17 (24)	Malassezia globosa 9 (13)	Cutaneotrichosporon haglerorum 3 (4)	Cutaneotrichosporon haglerorum 3 (4)	Penicillium paradoxum 3 (4)	Poitrasia circinans 3 (4)	Paradendryphiella arenariae 8 (11)	Pseudocamarosporium piceae 7 (10)	Talaromyces wortmannii 7 (10)	No Identification 6 (8)	Penicillium fagi 5 (7)	Pseudopithomyces angolensis 4 (6)
Patient bedrails	Malassezia restricta 26 (67)	Malassezia globosa 18 (46)	Baeospora myosura 1 (3)	Baeospora myosura 1 (3)	Clavispora lusitaniae 1 (3)	Penicillium paradoxum 1 (3)	Candida glabrata 2 (5)	Botryotrichum domesticum 1 (3)	Talaromyces wortmannii 1 (3)	Candida glabrata 2 (5)	Dinemasporium sasae 1 (3)	Penicillium fuscoglaucum 1 (3)
Bed tower 3	Malassezia restricta 97 (39)	Malassezia globosa 57 (23)	Penicillium paradoxum 3 (1)	Penicillium paradoxum 3 (1)	Penicillium paradoxum 2 (1)	Botryobasidium botryosum 1 (0)	Talaromyces wortmannii 20 (8)	Paradendryphiella arenariae 7 (3)	Tintelnotia destructans 7 (3)	No Identification 12 (5)	Penicillium fuscoglaucum 12 (5)	Penicillium fimorum 5 (2)
Air	Malassezia restricta 15 (21)	Malassezia globosa 5 (7)	Cutaneotrichosporon haglerorum 2 (3)	Cutaneotrichosporon haglerorum 2 (3)	Penicillium paradoxum 2 (3)	Botryobasidium botryosum 1 (1)	Talaromyces wortmannii 4 (6)	–	–	Penicillium fimorum 2 (3)	Penicillium fuscoglaucum 2 (3)	–
Bathroom floor	Malassezia restricta 20 (44)	Malassezia globosa 10 (22)	Neophaeococcomyces oklahomaensis 2 (4)	Neophaeococcomyces oklahomaensis 2 (4)	Cutaneotrichosporon haglerorum 1 (2)	Juncaceicola alpina 1 (2)	Talaromyces wortmannii 5 (11)	Paradendryphiella arenariae 2 (4)	Tintelnotia destructans 2 (4)	Penicillium fuscoglaucum 3 (7)	Epicoccum phragmospora 2 (4)	Penicillium fimorum 2 (4)
Clean linen storage	Malassezia restricta 3 (30)	Botryosphaeria agaves 1 (10)	–	Botryosphaeria agaves 1 (10)	–	–	Zasmidium cellare 2 (20)	Mucor brunneogriseus 1 (10)	Pseudocamarosporium piceae 1 (10)	No Identification 3 (30)	Cladosporium cycadicola 1 (10)	Penicillium fuscoglaucum 1 (10)
Dirty linen storage	Clavispora lusitaniae 1 (10)	Rhizopus americanus 1 (10)	–	Clavispora lusitaniae 1 (10)	Rhizopus americanus 1 (10)	–	Aspergillus niger 2 (20)	Talaromyces wortmannii 2 (20)	Tintelnotia destructans 2 (20)	No Identification 3 (30)	Aspergillus awamori 2 (20)	Candida albicans 1 (10)
HVAC exhaust vent	Malassezia restricta 26 (35)	Malassezia globosa 17 (23)	Penicillium paradoxum 3 (4)	Penicillium paradoxum 3 (4)	Vishniacozyma victoriae 2 (3)	Baeospora myosura 1 (1)	Talaromyces wortmannii 6 (8)	Paradendryphiella arenariae 5 (7)	Aspergillus niger 2 (3)	Penicillium fuscoglaucum 3 (4)	No Identification 3 (4)	Aspergillus awamori 2 (3)
Patient bedrails	Malassezia restricta 33 (83)	Malassezia globosa 25 (63)	Poitrasia circinans 2 (5)	Poitrasia circinans 2 (5)	Anguillospora rubescens 1 (3)	Clavispora lusitaniae 1 (3)	Talaromyces wortmannii 3 (8)	Candida albicans 2 (5)	Ambrosiella roeperi 1 (3)	Candida albicans 2 (5)	Penicillium fuscoglaucum 2 (5)	No Identification 2 (5)

### Identified genera over time

Across the 10-month sampling period, Malassezia dominated detections by direct-from-sample 18S qPCR, accounting for the majority of genera identified each month. This trend persisted consistently, limiting the visible diversity of other genera in the data. When Malassezia was excluded, a broader spectrum of genera emerged, with Penicillium and Mucor appearing more prominently (Supplemental Figure 1).

For culture-18S, Talaromyces was the most consistently identified genus over the study period, with a noticeable presence in nearly every month (Supplemental Figure 2). Candida and Mucor also appeared frequently, though their presence fluctuated. The monthly variability in genera suggests potential influence from seasonal or environmental conditions but no obvious patterns were discovered.

Culture-ITS showed the most diverse genus profile across time (Supplementary Figure 2). Penicillium was persistently identified each month, often as the most dominant genus, reaffirming its role as a ubiquitous environmental contaminant. Candida appeared frequently in early fall (September–November) and spring (March–May).

## Discussion

In this longitudinal, multi-unit and bed tower analysis of environmental fungal contamination in a large academic hospital, we found that direct-from-sample 18S qPCR detected fungi in more than twice as many samples as traditional culture methods. Despite qPCRs higher sensitivity for the detection of any fungi, each method had unique advantages and disadvantages identifying specific fungi. For clinically relevant fungi, qPCR more frequently identified *Mucor*, *Cutaneotrichosporon*, and *Penicillum* genera compared to culture methods, while culture methods identified a broader array of genera overall, including those frequently undetected by qPCR (*Talaromyces*, *Candida*, and *Aspergillus*). The differing predominance of Talaromyces in culture-18S results and Penicillium in culture-ITS results may reflect technical differences in taxonomic assignment between 18S and ITS sequencing. Also, the overwhelming abundance of *Malassezia* in the qPCR results reduced interpretability by masking other lower-abundance genera. The predominance of Malassezia in qPCR results may also reflect poor recovery of lipid-dependent Malassezia species on standard fungal culture media without lipid supplementation. Future fungal surveillance may benefit from combining broad fungal detection with species- or genus-specific primers targeting clinically important fungi

Fungal detection patterns also varied by building age, unit type, and sampling location. The oldest bed tower (BT1) showed the highest overall contamination by both qPCR and culture, while the newest (BT3) had the lowest, suggesting that infrastructure age, ventilation design, and materials may have influenced baseline fungal burden, consistent with previous findings.^13^ Although the three study bed towers had generally similar inpatient ventilation and filtration systems within standard inpatient care specifications, detailed engineering parameters such as MERV ratings and air exchange rates were not collected as part of this study and building-level differences should therefore be interpreted cautiously. Across units, contamination patterns were similar, but intensive care areas tended to have slightly higher positivity than general wards, possibly reflecting greater equipment density. At the sample level, bathroom floors, HVAC exhaust vents, and dirty linen storage were consistently the most contaminated sites, reinforcing their role as persistent environmental reservoirs across hospital environments.

To our knowledge, this is the first study to longitudinally compare molecular and culture-based fungal detection methods in a healthcare setting. By pairing traditional culture methods with direct-from-sample 18S qPCR and sequencing, this study provides a real-world assessment of each method’s strengths and weaknesses in detecting fungal contamination and identifying clinically relevant fungi. Importantly, Our culture-based methods likely represent an optimized estimate of culture performance because all isolates underwent molecular identification using 18S or ITS sequencing. As a result, the Culture–18S and Culture–ITS methods used here likely outperform standard culture-based identification used in many environmental surveillance settings. It also establishes a generalizable baseline for fungal contamination in healthcare settings via the inclusion of buildings of varying age, units of varying acuity, and both previously studied and novel fomites. Consequently, few comparable studies exist, making it difficult to contextualize our findings within the broader fungal surveillance literature. A recent multicenter study by García-Gutiérrez et al used conventional culture and ITS sequencing to survey fungal contamination across hospital zones, including air, HVAC, and surface sites similar to those sampled in our study using sponges.^16^ They reported *Cladosporium*, *Penicillium*, and *Aspergillus* as dominant genera and observed gradients in contamination linked to infrastructure and air protection levels, consistent with our findings across bed towers of differing age. Another study by Méheust et al comparing a novel detection method called solid-phase cytometry to standard culture found higher sensitivity for air samples but no advantage for surfaces. These results differ from our findings, as qPCR outperformed culture across all sample types in our study, including air, surfaces, and HVAC sites.^17^ These differences could be due to the differences in viability or spore detection as solid-phase cytometry detects only viable cells and does not detect non-metabolically active spores. Additionally, Méheust et al quantified all fungi and did not identify specific genera or species making comparisons difficult.

This study has limitations. First, cross-sectional monthly sampling may not represent true fungal contamination due to acute contamination events and the stochasticity of environmental contamination. However, since no patients in study units had an invasive fungal infection the potential influence of outbreak-related contamination is likely minimal. Second, qPCR detects genomic material rather than viability, so its higher sensitivity may be inflated by the presence of non-viable organisms. To address this, we focused on consistent spatial and temporal patterns across sampling events, which are less likely to reflect transient DNA contamination. Third, culture-based methods for fungi are highly variable compared to other microorganisms such as bacteria. The methods used in this study are appropriate to detect a wide range of fungi but are biased toward fast-growing species. However, these methods were applied uniformly across all samples to reduce bias from culture conditions. Additional limitations include the single-center design, which may limit generalizability despite enrolling units from three different buildings, and the lack of concurrent environmental parameters (humidity, air exchange rates, etc.) that could further contextualize variation in fungal burden.

In summary, this study demonstrates that molecular and culture-based fungal detection methods yield complementary but distinct insights into hospital environmental contamination. Although qPCR provided greater sensitivity and improved detection for several clinically relevant fungi, culture methods captured a broader diversity of viable organisms, including taxa not consistently identified by qPCR. These findings show promise for incorporating molecular detection into environmental fungal surveillance but also highlight the need for additional studies to refine assay design, establish interpretive thresholds, and validate clinical relevance. Future work should evaluate qPCR performance using species- or genus-specific primers, assess reproducibility across institutions and environmental conditions, and determine how results derived from molecular methods correlate with patient risk. Continued investigation will be critical to advance qPCR from an investigational research tool to a reliable component of infection prevention and hospital environmental monitoring programs.

## Supporting information

10.1017/ice.2026.10491.sm001 Warren et al. supplementary material Warren et al. supplementary material

## Supplementary material

The supplementary material for this article can be found at https://doi.org/10.1017/ice.2026.10491.

## Figure 1. Long description

The diagram illustrates the workflow for detecting fungi through culture-based and direct-from-sample molecular methods. The process begins with a sample collection using a swab, followed by DNA extraction. The extracted DNA undergoes 18S quantitative polymerase chain reaction (qPCR) for quantification. The culture method involves growing fungi on a petri dish, followed by 18S polymerase chain reaction (PCR) and internal transcribed spacer (ITS) sequencing. Sanger sequencing is then performed, and the results are analyzed using Nucleotide BLAST. For the direct-from-sample molecular method, next-generation sequencing is conducted, and the data is analyzed using SmartGene software to identify genus and species and determine relative abundances.

Navigate back to Figure 1..

## Table 1. Long description

The table presents data on fungi detection rates in hospital environments using two methods: direct-from-sample 18S qPCR and culture. It includes overall detection rates and specific rates for different sample locations such as air, bathroom floors, clean linen storage, dirty linen storage, HVAC exhaust vents, and patient bedrails. The table is divided into three bed towers, each with its own set of sample locations. The qPCR method generally shows higher detection rates compared to the culture method. For instance, air samples were positive in 72 cases (35%) by qPCR but only 6 (3%) by culture. Similar trends are observed for bathroom floors, HVAC exhaust vents, and patient bedrails. In contrast, detection rates for clean and dirty linen storage areas are more comparable between the two methods. The table highlights that bathroom floors, dirty linen storage, and HVAC exhaust vents are the most frequently contaminated sites according to both detection methods.

Navigate back to Table 1..

## Table 2. Long description

The table presents data on the level of phylogenetic identification via direct-from-sample 18S qPCR of hospital environmental samples positive for fungal presence. It includes 787 fungal detections resolved to at least the family, genus, or species level. The table has 20 rows and 4 columns, with headers for Family, Genus, and Species, each showing the number and percentage of detections. Key trends include the highest detections at the species level (536, 68%), followed by the genus level (145, 18%), and the family level (106, 13%). The data is further broken down by sample types such as Air, Bathroom floor, Clean linen storage, Dirty linen storage, HVAC exhaust vent, Patient bedrails, and specific bed towers. Notable patterns include higher species-level detections in Patient bedrails and Bed tower samples.

Navigate back to Table 2..

## Figure 2. Long description

The image contains four pie charts labeled a, b, c, and d, each representing different detection methods for identifying fungal genera in hospital environmental samples. Chart a shows the distribution using direct-from-sample 18S qPCR, with Malassezia at seventy percent, Mucor at five percent, Cutaneotrichosporon at three percent, Penicillium at two percent, Candida at one percent, Cladosporium at one percent, Aspergillus at one percent, Alternaria at one percent, Talaromyces at one percent, and other genera at fifteen percent. Chart b excludes Malassezia species, showing Mucor at seventeen percent, Cutaneotrichosporon at eleven percent, Penicillium at five percent, Candida at five percent, Talaromyces at four percent, Alternaria at three percent, Cladosporium at two percent, Aspergillus at two percent, Epicoccum at two percent, and other genera at forty-nine percent. Chart c represents culture followed by 18S, with Talaromyces at twenty percent, Candida at eleven percent, Paradendryphiella at ten percent, Tintelnotia at ten percent, Aspergillus at eight percent, Zasmidium at eight percent, Pseudocamarosporium at seven percent, Stenella at four percent, Mucor at five percent, and other genera at seventeen percent. Chart d shows culture followed by ITS, with Penicillium at twenty-one percent, no identification at fourteen percent, Candida at eleven percent, Alternaria at five percent, Talaromyces at four percent, Curvularia at four percent, Cladosporium at three percent, Epicoccum at three percent, Mucor at three percent, and other genera at thirty-two percent.

Navigate back to Figure 2..

## Table 3. Long description

The table presents data on the most common genera identified in hospital environmental samples positive for fungi using direct-from-sample 18S qPCR and culture detection methods. It includes columns for 18S qPCR results, 18S qPCR sans Mucorales spp., and culture via 18S and ITS, each detailing the most common genera and their frequencies. The table has 18 rows and 12 columns, with headers indicating the detection methods and the most common genera identified. Notable trends include the prevalence of Malassezia across all methods, with Mucor, Cutaneotrichosporon, and Penicillium being the next most common genera when Malassezia is excluded. The data highlights the distribution of these genera across different environmental samples, such as air, bathroom floor, clean linen storage, dirty linen storage, HVAC exhaust vent, patient bedrails, and bed tower. Each row provides specific details on the genera detected in various hospital locations, offering insights into fungal presence and potential clinical relevance.

Navigate back to Table 3..

## Table 4. Long description

The table compares the most common fungi species identified in hospital environmental samples using direct-from-sample 18S qPCR and culture detection methods. It includes data for 1st, 2nd, and 3rd most common species across various categories such as overall, patient room, bathroom, and public areas. The table lists specific fungi species and their prevalence in different contexts, highlighting variations in detection methods. Key trends and comparisons are noted, such as the dominance of certain species in specific environments.

Navigate back to Table 4..
