# Supplementary material for: Comparing rapid molecular and culture methods for detecting fungal contamination in healthcare environments
Source: Infect Control Hosp Epidemiol. 2026 Jun 18:1–10. Online ahead of print. doi: 10.1017/ice.2026.10491 (PMC13311461; doi:10.1017/ice.2026.10491)

**Supplemental Figure 1**. Identified Genera in Hospital Environmental Samples Positive for Fungi via 18s qPCR Over Time Including *Malassezia spp*. (Left) and Excluding *Malassezia spp*. (Right)


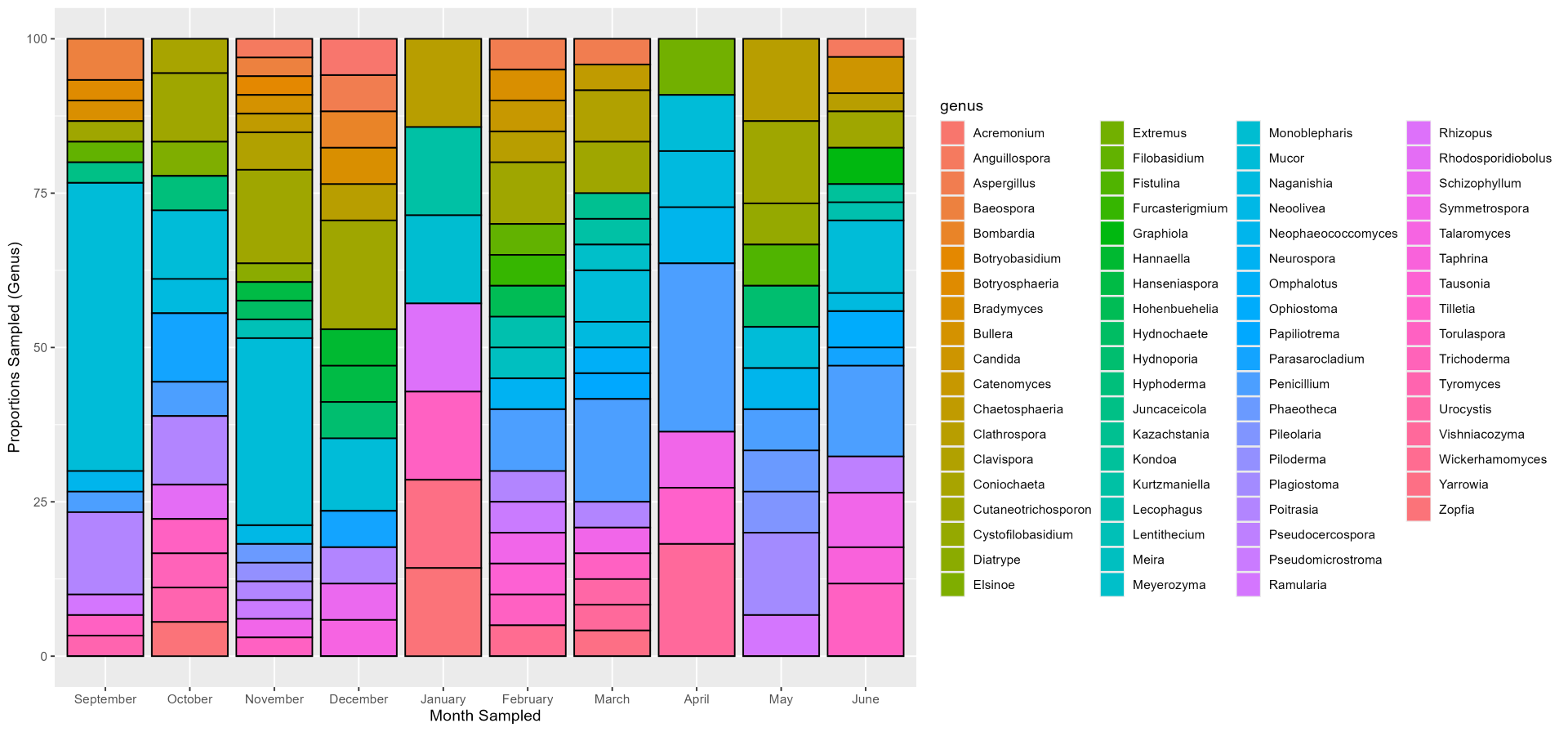


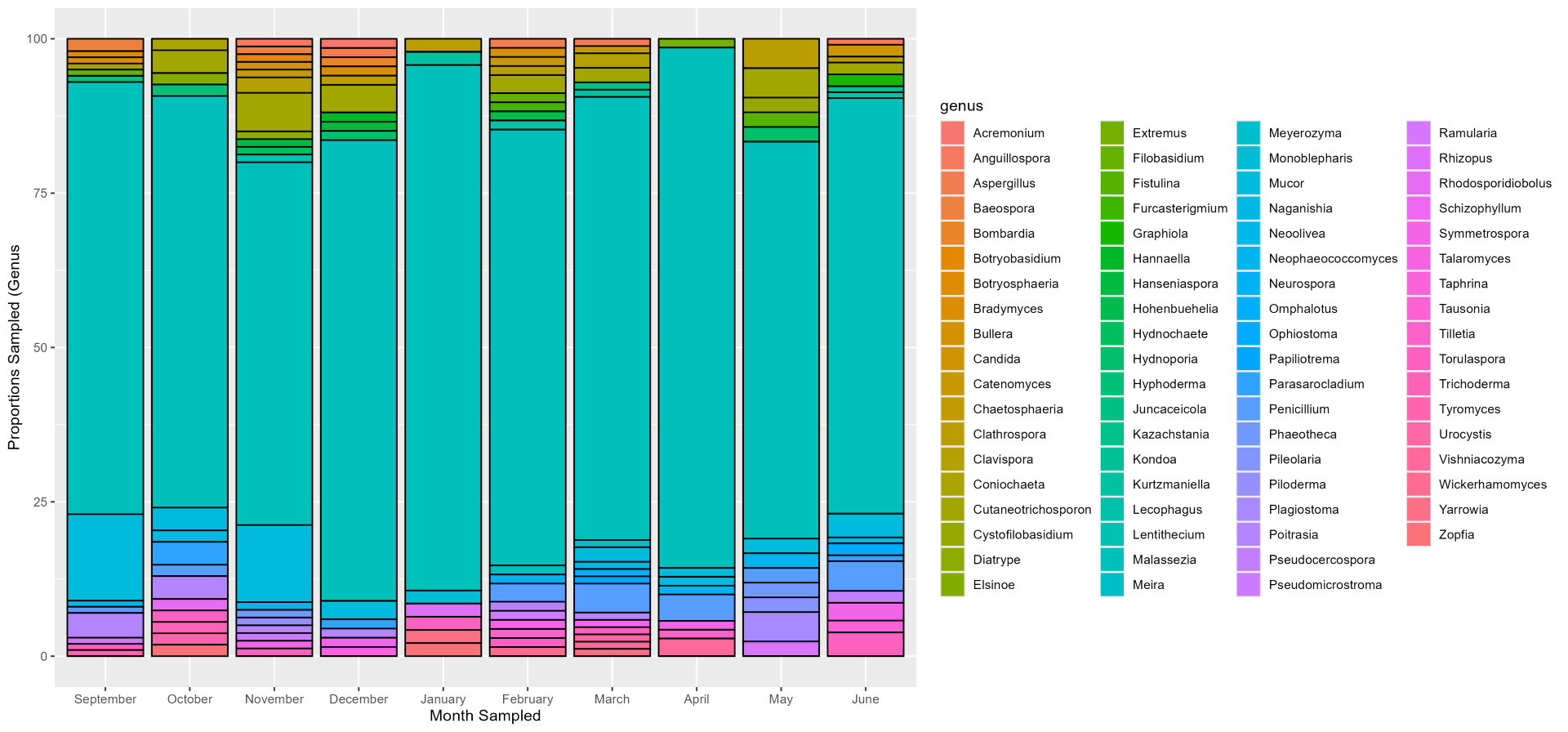
**Supplemental Figure 2**. Identified Genera in Hospital Environmental Samples Positive for Fungi via Culture Over Time via 18S (Left) and ITS (Right)


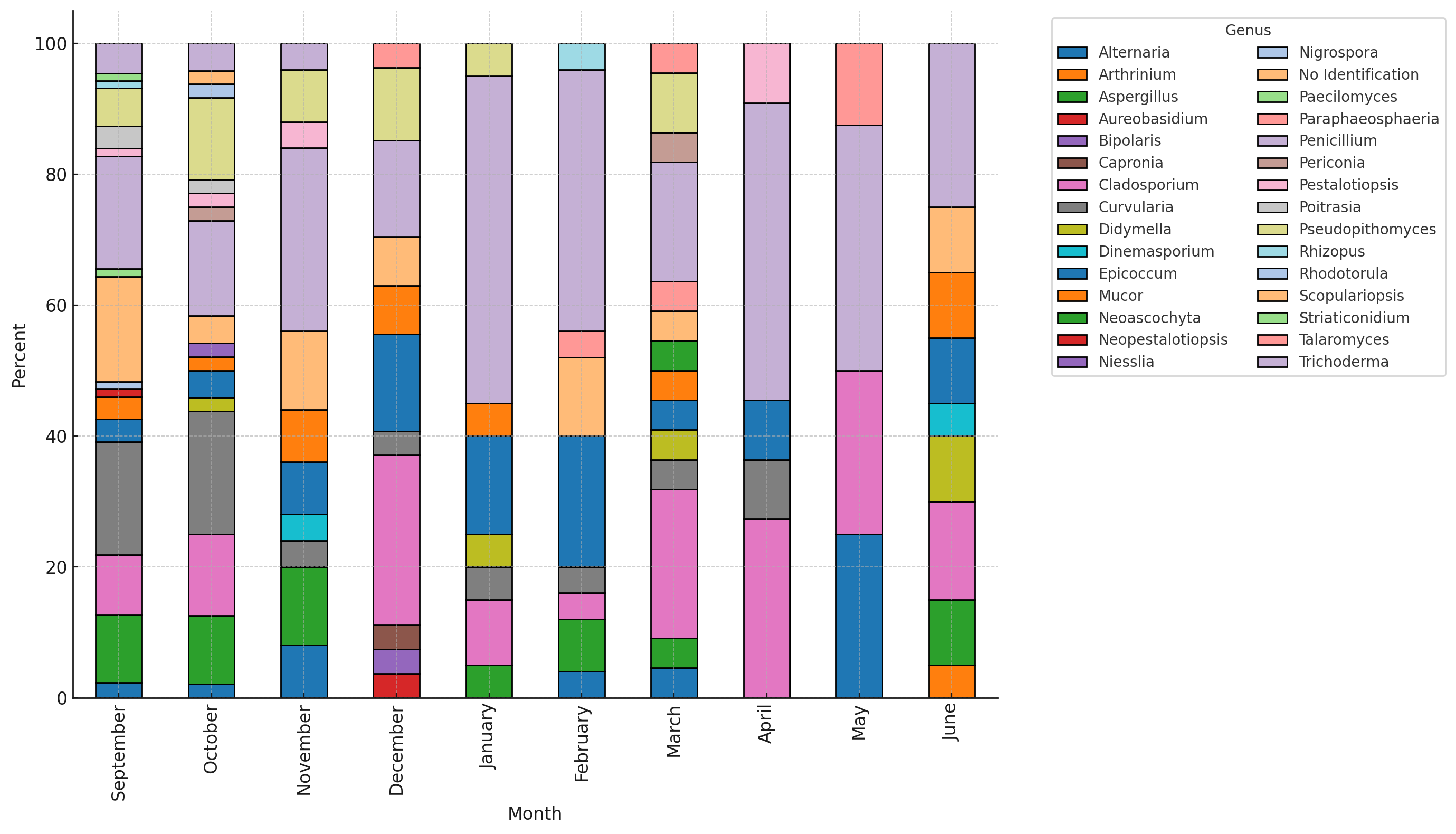

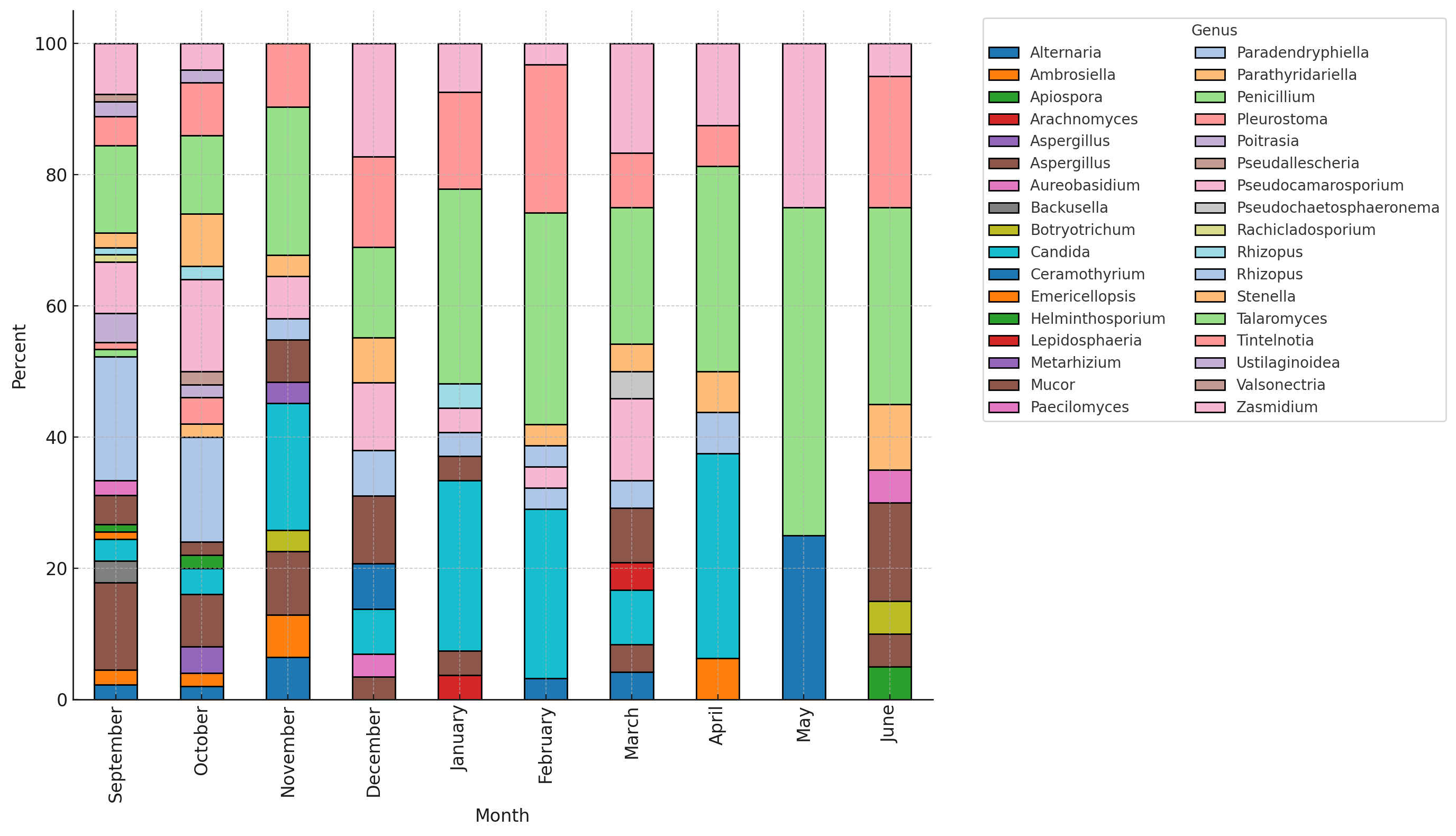

Supplement: Warren et al. supplementary material [file S0899823X26104917sup001.docx]
